# New Chemotypes for the Inhibition of (p)ppGpp Synthesis in the Quest for New Antimicrobial Compounds

**DOI:** 10.3390/molecules27103097

**Published:** 2022-05-12

**Authors:** Crescenzo Coppa, Luca Sorrentino, Monica Civera, Marco Minneci, Francesca Vasile, Sara Sattin

**Affiliations:** Dipartimento di Chimica, Università degli Studi di Milano, Via C. Golgi, 19, 20133 Milano, Italy; crescenzo.coppa@unimi.it (C.C.); luca.sorrentino@unimi.it (L.S.); monica.civera@unimi.it (M.C.); marco.minneci@unimi.it (M.M.); francesca.vasile@unimi.it (F.V.)

**Keywords:** AMR, persisters, (p)ppGpp, fragment screening, thermal shift assay, STD-NMR

## Abstract

Antimicrobial resistance (AMR) poses a serious threat to our society from both the medical and economic point of view, while the antibiotic discovery pipeline has been dwindling over the last decades. Targeting non-essential bacterial pathways, such as those leading to antibiotic persistence, a bacterial bet-hedging strategy, will lead to new molecular entities displaying low selective pressure, thereby reducing the insurgence of AMR. Here, we describe a way to target (p)ppGpp (guanosine tetra- or penta-phosphate) signaling, a non-essential pathway involved in the formation of persisters, with a structure-based approach. A superfamily of enzymes called RSH (RelA/SpoT Homolog) regulates the intracellular levels of this alarmone. We virtually screened several fragment libraries against the (p)ppGpp synthetase domain of our RSH chosen model Rel*_Seq_*, selected three main chemotypes, and measured their interaction with Rel*_Seq_* by thermal shift assay and STD-NMR. Most of the tested fragments are selective for the synthetase domain, allowing us to select the aminobenzoic acid scaffold as a hit for lead development.

## 1. Introduction

The rising of bacteria resistant to the currently available antibiotic arsenal is posing a serious threat to the way of life we have become accustomed to over the past century since Fleming isolated and characterized penicillin [1,2]. If left unchecked, antimicrobial resistance (AMR), paired with the decreasing number of new antibiotics progressing through the clinical pipeline, will lead to millions of deaths each year in a few decades [3].

AMR manifests itself in stable, heritable genetic forms, as well as in lesser-known transient phenotypes that are more elusive and difficult to recognize and tackle [4]. On the other hand, antimicrobial compounds currently in the clinical phase consist mostly of derivatives of established classes, with an urgent need for drugs that address multidrug-resistant Gram-negative bacteria [3].

The search for new chemical entities, targeting non-essential pathways that play a role in both the infection process (e.g., bacterial adhesion, quorum sensing, virulence, and biofilm formation) and the insurgence of genetic resistance, is an attractive strategy for attaining new antimicrobial drugs exerting minimal selective pressure. 

We posit (p)ppGpp-signalling molecules (guanosine tetra- or penta-phosphate) [5] as key players in both processes [6]. Indeed, (p)ppGpp is a ubiquitous alarmone that directs bacterial adaptation to environmental changes (e.g., nutrient starvation, oxidative stress, etc.) by binding various targets involved, e.g., in nucleotide metabolism, DNA replication and repair [7], transcription [8], and translation [9,10]. This alarmone thus has pleiotropic effects on bacterial cell physiology [11], regulating cell size, virulence, quorum sensing, and biofilm formation [12,13,14,15]. In particular, its ability to downregulate cell metabolism and cell growth hints at its role in the insurgence of the bacterial non-heritable dormant phenotype called persister [16]. Persisters are transiently tolerant to antibiotic treatment (i.e., they are a form of phenotypic AMR) until they revert to the awake state and resume growth, constituting an infection reservoir that sustains chronic and recurrent infections and paves the way to the acquisition of genetic resistance [17,18]. Over the last two decades, extensive research has revealed different molecular mechanisms leading to their formation [19], and one of them is the accumulation of (p)ppGpp together with its downstream effects.

**Figure 1:**
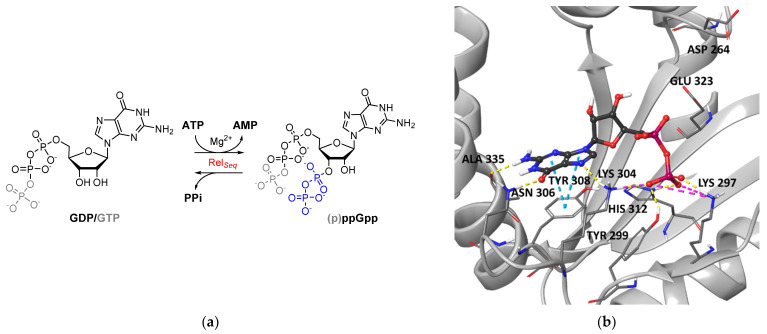
(**a**) Enzymatic reaction that regulates intracellular (p)ppGpp levels catalyzed by RSH enzymes such as Rel*_Seq_*; (**b**) X-ray structure of GDP in the SYNTH site of Rel*_Seq_* (1VJ7, chainA). GDP is represented in ball and stick, and the key interacting amino acids are labeled.

Intracellular (p)ppGpp levels are regulated by enzymes belonging to the RSH (RelA/SpoT Homolog) superfamily [20]. These enzymes catalyze (p)ppGpp synthesis via a Mg^2+^-dependent pyrophosphate transfer from ATP to the 3′-OH group of either GDP or GTP. They also catalyze (p)ppGpp hydrolysis leading to the release of pyrophosphate (PPi) using distinct active sites in different protein domains (Figure 1a). “Short” RSH proteins harbor either only the synthetase (SAS, small alarmone synthetases) or hydrolase (SAH, small alarmone hydrolases) domain, respectively. “Long” RSH proteins contain both catalytic domains and a C-terminal regulatory domain (CTD) that activates alarmone synthesis by promoting Rel oligomerization [21] and/or upon binding to stalled ribosomes (i.e., ribosomes bound to uncharged tRNAs during aminoacid starvation) [22,23] or favors alarmone hydrolysis by directly inhibiting the synthetase site [24]. In addition, reciprocal regulation of the two catalytic domains has been postulated with mechanisms that vary among species [25,26,27,28].

The search for inhibitors of Rel enzymes synthetase activity dates back to the early 2010s, when Relacin [29] and a few other (p)ppGpp analogs [30,31] were described by Wexselblatt and co-workers. In all cases, IC_50_ values measured against RelA (*E. coli*) and Rel from *D. radiodurans* were estimated to be between 1 and 5 mM, with relatively low ligand efficiency and no subsequent further optimization reported. More recently, extensive efforts, including high-throughput screening (Rel*_Bs_*, *B. subtilis*) and an expanded library of (p)ppGpp analogs (RelA), have identified some low µM, non-specific Rel inhibitors [32,33], while screening of a large pharmaceutical library (GSK, >2 M compounds) for inhibitors of Rel*_Mtb_* (*M. tuberculosis*) identified only one compound (X9) as a potential lead for a combination TB therapy with isoniazid [34]. 

In this framework, we optimized the synthesis of a fluorescent (p)ppGpp selective chemosensor [35], and here, we report the identification of three novel chemical scaffolds for the design of selective RSH inhibitors through fragments virtual screening campaigns followed by experimental validation of representative fragments on the synthetase site of the “long” RSH protein Rel*_Seq_* (*S. equisimilis*).

## 2. Results and Discussion

### 2.1. Fragment Libraries Virtual Screening in Rel_Seq_ Synthetase Site

We chose as a protein model the X-ray crystal structure reported for Rel*_Seq_* in a so-called synthetase-ON conformation, i.e., the chain A of the pdb structure 1VJ7 (residues 1–385), which carries the GDP substrate in the synthetase catalytic site [25]. This is a truncated form of the protein, lacking the C-terminal regulatory domain. 

By analyzing the interactions of GDP within the catalytic site (Figure 1b), we could observe that it binds to the G-loop (Tyr299-Ser310), forming a π–π stacking interaction with the side chain of Tyr308 through its guanine ring. H-bonds with the side chains of Lys304 and Asn306 and with the backbone of Ala335 stabilize this core interaction. In addition, the GDP pyrophosphate moiety forms salt bridges with Lys304 and Lys297 side chains and H-bonds with Tyr299 and His312 side chains. 

Our analysis revealed that the Rel*_Seq_* synthetase-ON catalytic site conformation could not be catalytically competent, as it lacks the space necessary to accommodate the pyrophosphate donor ATP, and the reported catalytic residues Asp264 and Glu323 are unfavorably oriented to promote the reaction (Figure 1b). On this basis, we constructed and reported a catalytically competent Rel*_Seq_* chimera model based on the structure of the SAS RelP from *S. aureus*, where the resulting catalytic site is considerably more extended [36]. However, we performed an initial virtual screening on the Rel*_Seq_* X-ray crystal structure in order to focus our binding site exploration on the region occupied by the enzyme substrate GDP.

During protein preparation (1VJ7, chain A, see Section 3), we took a closer look at the protonation state of His312, interacting with the β-phosphate group of GDP in the crystal. Although the neutral form should be more plausible, given the pH working conditions of the enzyme (activity usually tested at pH 7–9), we decided to generate a model with the residue in the protonated form as well (Hip312) in order to assess the validity of this assumption. We set up and validated a docking protocol within the synthetase binding site for both models by re-docking the co-crystallized GDP molecule using GLIDE v8.0 (Supplementary Figures S1 and S2). [37] 

**Figure 2:**
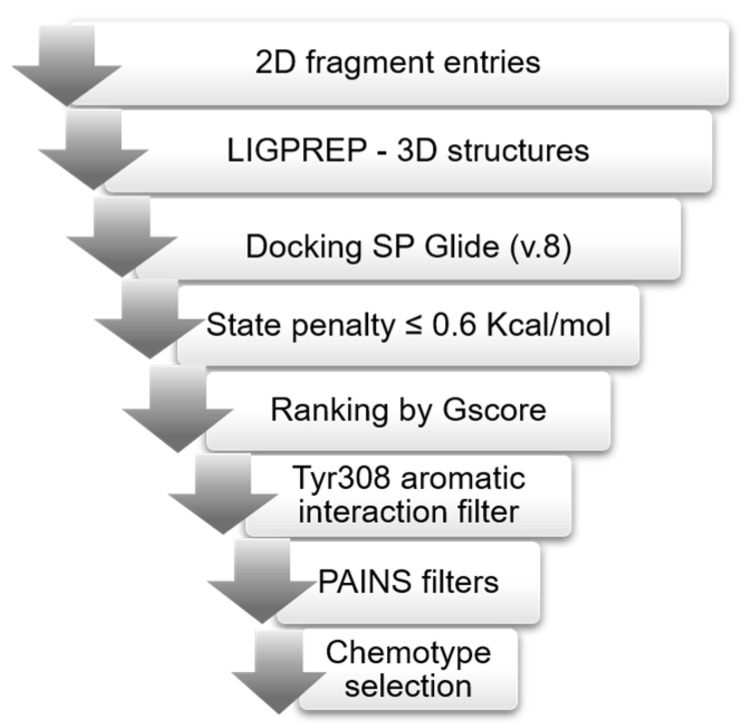
Virtual screening workflow. Ligprep generated 3D structure from 2D fragments; Glide docked the 3D structures into both Rel*_Seq_* models (His312 and Hip312). A state penalty filter excluded unfavorable states, and the resulting docking poses were ranked according to Gscore. Only fragments forming an aromatic interaction with Tyr308 were retained, and after removing PAINS, we evaluated the top 1% ranked poses identifying the most representative chemotypes.

Several chemical vendors currently make available virtual structure datasets of fragment libraries, often organized according to specific experimental properties (e.g., solubility). In order to maximize the chemical space explored in our screening [38,39], we selected seven different libraries of commercially available fragments, *Maybridge* Rule of 3, *Asinex* Fragments, *Life Chemicals* Fragment Library with Experimental Solubility Data I and II, *OTAVA* Solubility fragment library, *Chembridge* Fragment library, and *SPECS* fragment library, amounting to a total of 58,321 2D entries (see Section 3 and Supplementary Table S1). We implemented the validated docking protocol for the virtual screening (VS) of the selected fragment libraries following the workflow shown in Figure 2. For each library, Ligprep [40] converted 2D entries into 3D structures considering stereoisomers, tautomers, and protonation states, leading to an increase in the total number of structures up to 114,966.

### 2.2. Post-Docking Analysis and Chemotype Selection

**Figure 3:**
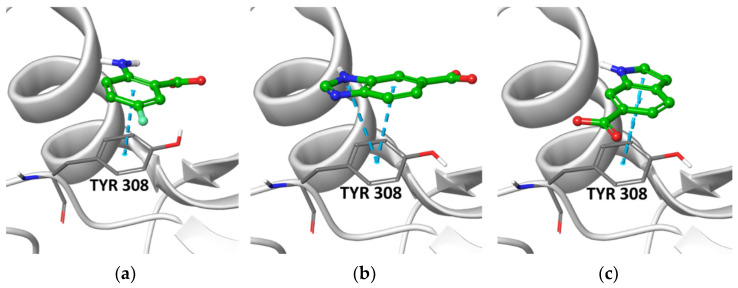
Best poses of representative structures of the three chemotypes selected for experimental validation. (**a**) aminobenzoic acid **A3** docked in the Hip312 grid; (**b**) Benzimidazole **B2** docked in the His312 grid; (**c**) indole **I1** docked in the His312 grid. Fragments are shown as balls and sticks with green carbon atoms. The protein is shown in grey, with the side chain of Tyr308 highlighted.

We removed from the docking outputs (one pose saved for each fragment) the less stable tautomeric and ionization forms, as determined by Epik [41] (state penalty value ≤ 0.6 kcal/mol). We removed duplicates from the merged outputs and ranked them by Gscore. Finally, we applied an aromatic interaction filter with Tyr308 (i.e., an aromatic ligand atom must be within 5 Å from any heavy atom of the Tyr308 side chain), a key residue that, when mutated into asparagine or serine, inhibits the synthetic activity of the enzyme, and removed PAINS (pan-assay interference compounds) using the filters provided by Canvas [42,43] in order to exclude frequent hitters [44]. This work resulted in the selection of 30,126 and 30,960 fragments for the His312 and Hip312 grids, respectively. We visually inspected and assessed the top 1% of docked poses, identifying three recurrent chemotypes, i.e., benzimidazole, aminobenzoic acid, and indole (for the calculated enrichment factors, see Supplementary Table S2) and three singletons (the best pose of representative fragments is shown in Figure 3 and Supplementary Figure S3). Aminobenzoic acids emerged mainly from the Hip312 model and benzimidazoles mainly from the His312 model, while indoles emerged from both to a lesser extent.

**Figure 4:**
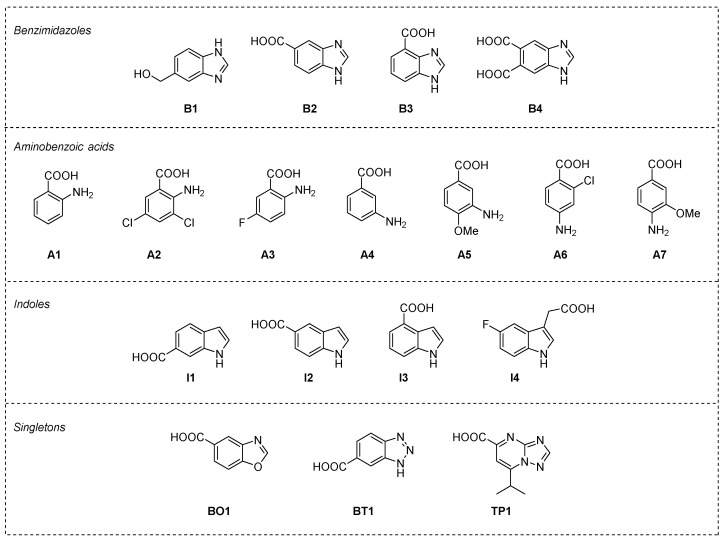
Fragments selected for experimental validation (i.e., thermal shift assay) grouped by chemotype: benzimidazoles (**B1**–**B4**), aminobenzoic acids (**A1**–**A7**), indoles (**I1**–**I4**), and the three singletons (**BO1**, **BT1,** and **TP1**).

In order to maximize the chemical space explored, we performed a similarity search (Tanimoto index ≥ 90%) using the PubChem database [45] and the representative structures of each chemotype as input. We applied the same screening workflow (see Supplementary Information) to the expanded set of fragments leading to the final selection of eighteen fragments for experimental validation (Figure 4). 

Prior to conducting biochemical assays, we assessed the docking poses stability by running molecular dynamics (MD) simulations. Starting from the best pose for each fragment we ran 100 ns simulations using Desmond [46] (NPT, T = 300 K, p = 1 atm, TIP3P [47], OPLS3e [48], dt = 2 fs). We considered the aromatic interaction with Tyr308 described above as the key feature to be maintained and monitored during the simulations. All the examined fragments form stable interactions with the residues involved in the binding of the GDP guanine ring in the X-ray structure, retaining, in particular, stable contact with the side chain of Tyr308, with the exception of fragments B1 and I2 that exit the binding pocket (Supplementary Figure S4). 

### 2.3. Thermal Shift Assay on Selected Fragments vs. Rel_Seq_ Protein Constructs

With the aim of studying protein–ligand interactions between Rel*_Seq_* constructs and selected fragments, we evaluated the use of different techniques, such as tryptophan assay, microscale thermophoresis (MST), isothermal calorimetry (ITC) or thermal shift assay (TSA). The tryptophan assay is based on ligand-induced conformational changes in the local environment surrounding tryptophan residues in the target protein. Irradiation at 280 nm is followed by the detection of tryptophan fluorescence emission at different wavelengths. We used this technique to measure the Kd values of the natural substrates GDP and ATP (see below), but it is not applicable in the case of fragments due to their high absorbance at 280 nm. On the other hand, the currently available protein-labeling reagents for MST were not compatible with our protein constructs. We chose TSA over ITC due to its higher potential throughput and lower amount of protein required.

Thermal shift is an experimental technique in which protein thermal denaturation is monitored following the increase in fluorescence reported by a protein-bound dye [49]. In particular, an environment-sensitive hydrophobic dye (e.g., SYPRO Orange) binds to hydrophobic regions that become progressively exposed during thermal denaturation, resulting in an increase in its fluorescence emission. Since the binding of small molecules (e.g., fragments) to the protein can cause conformational changes that affect its melting temperature, this technique allows the screening of several compounds in a range of concentrations without consuming sizeable amounts of protein. 

We preliminary determined by TSA the dissociation constants (*K*_d_) of complexes engaged by Rel*_Seq_* with its natural substrates, ATP (0.49 ± 0.09 mM) and GDP (0.26 ± 0.06 mM), finding values comparable to those obtained by tryptophan assay (*K*_d_^ATP^ = 0.39 ± 0.04 mM, *K*_d_^GDP^ = 0.15 ± 0.01 mM) and confirming the robustness of this technique (Table 1, entries 1 and 2).

We therefore evaluated the affinity of the 18 fragments selected from our *in silico* screening for the bifunctional protein Rel*_Seq_* by titration of the protein in a thermal shift assay (Table 1). We initially used Rel*_Seq_* 1–385, a truncated construct lacking the abovementioned C-terminal regulatory domain with a catalytic activity intrinsically shifted towards (p)ppGpp synthesis (12-fold higher than the full-length protein) [50].

Interestingly, all but four of the tested fragments showed a dose-dependent interaction with the bifunctional protein with *K*_d_ values in the low millimolar range.

In particular, only one of the four tested benzimidazoles showed a measurable affinity for Rel*_Seq_* (1–385) (**B3**, Table 1, entry 5), whereas fragment **B2** yielded a biphasic curve that requires further investigation (Table 1, entry 4). All of the selected aminobenzoic acids showed good affinities for the protein, with the exception of **A2** (Table 1, entries 7–13), while among the four indoles tested, all interacted in a dose-dependent manner with Rel*_Seq_* (1–385) (Table 1, entries 14–17). Finally, two of the three singletons tested (**BO1** and **TP1**) showed a low mM affinity for the protein.

We assessed the fragments’ selectivity for Rel*_Seq_* synthetase domain by performing TSA experiments on two mono-functional truncated protein constructs: Rel*_Seq_* SYNTH (residues 79–385) and Rel*_Seq_* HYD (residues 1–224), presenting only the synthetase or hydrolase protein domain, respectively. Both constructs retain part of the central 3-helix bundle to ensure proper folding, especially in the case of the less stable SYNTH domain [50].

The four fragments that failed to show binding to the bifunctional protein (**B1**, **B4**, **A2**, and **BT1**) also failed to show dose-dependent effects on the two truncated constructs, while the biphasic curve initially observed for **B2** was determined to be a selective interaction with the HYD domain, with no affinity for the SYNTH domain.

With the exception of **B3**, which shows comparable affinities for both catalytic domains, all the fragments binding to Rel*_Seq_* (1–385) showed a remarkable selectivity for the SYNTH domain, even with a generally increased absolute value for the measured *K*_d_. This is probably due to the lower overall stability of the SYNTH domain compared to the full protein, as previously reported by Mechold et al. [50]. Indeed, Rel*_Seq_* SYNTH (79–385), despite being catalytically functional (data not shown), requires the use of a non-ionic surfactant in the purification steps in order to avoid precipitation. 

### 2.4. STD-NMR Protein–Fragment Interaction Experiments

The relatively weak affinity of the fragments measured by TSA directed us towards NMR methods to assess the specificity of the fragments-protein interactions. Indeed, ligand-based NMR methods [51] can be applied to weak and transient protein–ligand complexes that are difficult to study with other structural techniques and do not require protein labeling (since only NMR signals of the small molecule are detected), and only a small amount of protein is required. In particular, STD (Saturation Transfer Difference) exploits NOE effects between the protein and the ligand to map target–ligand interactions and to characterize biologically relevant complexes [52,53].

**Figure 5:**
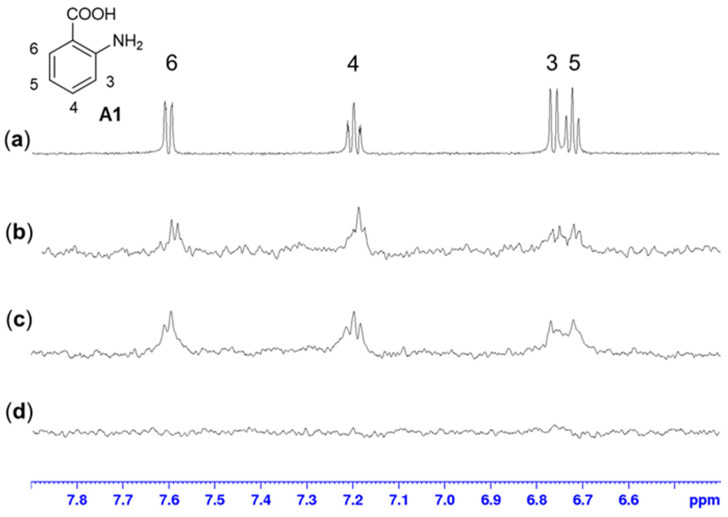
STD-NMR experiments (fragment:protein ratio 1000:1; fragment concentration 3 mM). (**a**) ^1^H-NMR of fragment **A1** in phosphate buffer at 298 K. (**b**) STD-NMR experiment of **A1** with Rel*_Seq_* (1–385). (**c**,**d**) STD-NMR experiment of **A1** with Rel*_Seq_* SYNTH (79–385) and with Rel*_Seq_* HYD (1–224), respectively. The same binding mode can be observed for full-length Rel*_Seq_* and Rel*_Seq_* SYNTH, while no binding can be detected with Rel*_Seq_* HYD.

Considering the promising selectivity profile of the aminobenzoic acids for the Rel*_Seq_* synthetase domain, we performed STD-NMR experiments with fragment **A1** as the representative chemotype. The results confirmed the binding event and showed a good interaction for the aromatic protons of **A1** with Rel*_Seq_* (1–385) (Figure 5b). Comparable overall STD intensities, suggesting a similar binding mode, were observed for Rel*_Seq_* SYNTH (79–385), confirming the specificity of the interaction (Figure 5c). On the other hand, the Rel*_Seq_* HYD (1–224) construct did not produce any magnetization transfer (Figure 5d). STD experiments performed on Rel*_Seq_* (1–385) with the two non-binding fragments **B1** and **A2** did not show any significant interaction, refuting artifacts or non-specific binding (Supplementary Figures S5 and S6, respectively).

## 3. Materials and Methods

### 3.1. Computational Methods

**Protein Preparation.** Rel*_Seq_* three-dimensional structure (PDB 1VJ7, chain A, residues 5–341) was prepared for docking calculations using the ‘Protein Preparation Wizard’ of Schrödinger^®^ suite [40] and OPLS_2005 force field [54]. All water molecules were deleted, and the gaps of the HD domain (K110-N123 and K153-D158) were built using Prime [55]. The residues’ protonation states were determined according to PROPKA at pH 7. Two models were built considering the two possible protonation states of His312, i.e., the neutral (His312) and the protonated (Hip312) form. According to Epik [41] results at pH 7± 2, the GDP molecule is fully deprotonated (total formal charge of −3). Hydrogen bonds were optimized according to the exhaustive sampling option, and the entire complexes were optimized by using a restrained minimization (root-mean-square deviation on heavy atoms < 0.30 Å). The K110-N123 and K153-D158 gaps of the HD domain were built, and the former was further refined using the ‘refine loops’ tool of Prime (OPLS3e [48], VSGB [56]) with default parameters. Five structures were generated, and the model with the lowest Prime energy was selected for the docking calculation.

**Docking protocol.** Grid-Based Ligand Docking With Energetics (Glide) [37] v.8.0 software was used with the OPLS_2005 force field. Receptor grids for HIS312 and HIP312 systems were generated in a cubic region (24.5 Å) centered on GDP molecules with an inner cubic box of 10 Å. The receptor was considered a rigid body, while the ligands were considered flexible. The standard precision (SP) method was applied with default parameters. No Epik state penalty was added to the Glide score. The docking protocol was validated for the X-ray ligand by saving five poses after a post-minimization of the first 10 poses. 

The top-ranked poses succeeded in reproducing the experimental binding mode of GDP (RMSD on heavy atoms of 0.78 Å and 1.23 Å in the HIS312 and HIP312 models, respectively) (Supplementary Figures S1 and S2). 

**Fragment libraries preparation.** Seven fragment libraries were downloaded:

- Maybridge Ro3 Diversity Set (2500 fragments) (www.maybridge.com, accessed on 9 June 2015);
- Asinex-Fragments-21872 (21,872 fragments) (www.asinex.com, accessed on 18 January 2019);
- ‘Fragment Libraries with Experimental Solubility Data’, two datasets from Life Chemicals (11,667 and 2921 fragments, respectively) (www.lifechemicals.com, accessed on 1 February 2019);
- OTAVA Solubility Fragment Library (1021 fragments) (www.otavachemicals.com, accessed on 4 February 2019);
- FragmentLibrary_sdf_13808 (13,808 fragments) from CHEMBRIDGE (www.chembridge.com, accessed on 18 April 2019)
- Preplated Fragment-Based Library (4532 fragments) from SPECS (www.specs.net, accessed on 11 February 2019).

For each fragment, we generated 3D structures, tautomers, stereoisomers (at most 32 per ligand), and protonation states (Epik at pH = 7 ± 2) using the Ligprep tool [40]. Their energy was minimized using ‘MacroModel’ [40], implemented with truncated Newton conjugated gradient method [57], and the resulting structures were used as input for docking calculations (see Supplementary Table S1).

**Molecular Dynamics simulations.** Molecular dynamics (MD) simulations (100 ns, NPT, OPLS3e [48], T = 300 K, Langevin thermostat [58] relaxation time = 1.0 ps; p = 1 atm; barostat relaxation time = 2.0 ps [59]) were carried out using Desmond [46] starting from the best pose of the eighteen fragments selected for the TSA (Hip312 best pose for aminobenzoic acids, His312 best pose for all the other fragments). Atomic coordinates were saved every 100 ps for a total of about 1000 frames. The systems were solvated into a (10 Å side) cubic box of TIP3P [47] water molecules and neutralized by adding Cl^−^ and Na^+^ ions at a physiological concentration of 0.15 M NaCl. The systems were equilibrated by applying the ‘desmond_npt_relax.msj’ protocol available in Desmond with default parameters. 

### 3.2. Experimental Methods

**Cloning, Expression, and Purification ofRel*_Seq_*constructs.** A pET21 expression vector containing the DNA sequence coding for Rel*_Seq_* 1–385 fused with a C-terminal His-tag was purchased from Giotto Biotech. Two truncated constructs of the bifunctional enzyme, Rel*_Seq_* 79–385 (Rel*_Seq_* SYNTH) and Rel*_Seq_* 1–224 (Rel*_Seq_* HYD) [50], presenting only the synthetase or hydrolase domain, respectively, were obtained with the Q5 Site-directed mutagenesis kit (New England Biolabs). Each protein construct was overproduced in BL21(DE3) *Escherichia coli* cells (Merck), grown in LB medium. Protein expression was induced by the addition of 0.5 mM IPTG and prolonged overnight at 25 °C for Rel*_Seq_* 1–385 and Rel*_Seq_* HYD and at 20 °C for Rel*_Seq_* SYNTH. 

In a typical purification, bacterial cells harvested by centrifugation were resuspended in lysis buffer (50 mM Tris-HCl pH 8.0, 250 mM NaCl, 10 mM imidazole, 0.5 mM TCEP), supplemented with 1 mM phenylmethanesulfonylfluoride, 20 μg/mL DNAse I (Merck) and, only in the case of Rel*_Seq_* SYNTH, 0.1% Triton X-100. Cell disruption was performed by sonication, and, after high-speed centrifugation and microfiltration, the resulting bacterial soluble extract was loaded on two 1 mL Ni Sepharose HisTrap columns (GE Healthcare), connected in series and equilibrated with lysis buffer. Elution of Rel*_Seq_* constructs was achieved by applying a linear gradient of elution buffer (50 mM Tris-HCl pH 8.0, 250 mM NaCl, 500 mM imidazole, 0.5 mM TCEP) over 15 column volumes. After a size exclusion chromatography step on a HiPrep 16/60 Sephacryl S-200 HR (GE Healthcare), Rel*_Seq_* constructs were stored at −80 °C in 50 mM Tris-HCl pH 8, 200 mM NaCl, 5% glycerol.

**Thermal shift assays on selected fragments vs. Rel*_Seq_* constructs.** The binding of the selected fragments to Rel*_Seq_* constructs was assessed by titration of the protein in thermal shift assays, performed using a Step One Real-Time PCR system (Thermo Fisher Scientific, Waltham, MA, USA). Assays were performed in 20 mM Tris-HCl, pH 8, containing 150 mM NaCl. The final protein concentration was kept at 0.5 mg/mL for all Rel*_Seq_* constructs, and the fluorescent Protein Thermal Shift Dye (Thermo Fisher Scientific, Waltham, MA, USA) was used to monitor protein unfolding within the excitation/emission ranges 470–505/540–700 nm. Each fragment was dissolved in DMSO at a stock concentration of 200 mM, and two-fold dilution series were prepared to have final compound concentrations ranging from 0.3 mM to 10 mM; 2.5% DMSO was added in place of the fragments for control samples. Assays were performed in triplicate at a final volume of 15 µL in MicroAmp™ Fast Optical 48-well reaction plates (Thermo Fisher Scientific, Waltham, MA, USA) sealed with adhesive films. Plates were heated from 25 to 90 °C with a heating rate of 0.5 °C/min. The *K*_d_ of protein–ligand complexes engaged by Rel*_Seq_* constructs with the tested fragments was calculated from the plot of protein melting temperature variations as a function of fragment concentrations with the equation for Ligand Binding (1 site) provided with the software GraFit 5.0 (Erithacus Software, Staines, UK).

**STD-NMR experiments on selected fragments vs. Rel*_Seq_* constructs.** Experiments were performed on a 600 MHz Bruker Avance spectrometer. All experiments were acquired at 298 and 283 K on the free ligands in deuterated phosphate buffer pH 7.4. A DMSO-d6 percentage of about 5% was added to dissolve the fragments. In 1D spectra, water suppression was achieved by excitation sculpting sequence. STD NMR experiments were performed using WATERGATE 3-9-19 pulse sequence for water suppression. On-resonance irradiation of the protein was performed at a chemical shift of −0.05 ppm and 10 ppm; off-resonance irradiation was applied at 200 ppm, where no protein signals were visible. Selective pre-saturation of the protein was achieved by a train of Gauss-shaped pulses of 49 ms in length each. STD spectra were acquired with a saturation time of 2.94 s for all compounds. Blank experiments were conducted in the absence of protein in order to avoid artifacts. We tested several protein/fragment ratios (i.e., 1:100, 1:200, and 1:1000) and found that a 1:1000 ratio with a protein concentration of 3 µM (500 μL final volume) afforded the best signal-to-noise ratio.

## 4. Conclusions

In conclusion, we targeted the intracellular accumulation of (p)ppGpp, a bacterial stringent-response-signaling molecule involved in the insurgence of persisters, a form of phenotypic AMR, and in bacterial virulence. New chemical entities with antimicrobial activity targeting non-essential pathways, such as (p)ppGpp signaling, are urgently needed to fight and prevent antimicrobial resistance. 

We performed an extensive structure-based *in silico* fragment screening on the synthetase site of the bifunctional enzyme Rel*_Seq_*, selecting three main chemotypes. Protein–fragment interaction experiments evidenced several low mM affinity binders. In particular, the aminobenzoic acid scaffold showed a marked synthetase domain selectivity and was therefore selected for the rational design of enzyme inhibitors that will be described in due course. Potent and selective Rel inhibitors will enable to shed light on the role of (p)ppGpp signaling in persisters’ formation and pave the way to low-selective-pressure antimicrobial therapeutic approaches.

## Figures and Tables

**Table 1:** Thermal shift assay. *K*_d_ (mM) of protein–ligand complexes engaged by Rel*_Seq_* constructs with the tested fragments.

Entry	Compound	*K*_d_ Rel*_Seq_* 1–385	*K*_d_ Rel*_Seq_* SYNTH	*K*_d_ Rel*_Seq_* HYD
1	**ATP**	0.49 ± 0.09 (0.39 ± 0.04) ^1^		
2	**GDP**	0.26 ± 0.06 (0.15 ± 0.01) ^1^		
3	**B1**	*No binding*	*No binding*	*No binding*
4	**B2**	*Biphasic*	*No binding*	1.9 ± 0.7
5	**B3**	3.4 ± 0.3	4.3 ± 0.4	3.3 ± 0.9
6	**B4**	*No binding*	*No binding*	*No binding*
7	**A1**	1.2 ± 0.3	10.8 ± 2.2	*No binding*
8	**A2**	*No binding*	*No binding*	*No binding*
9	**A3**	1.5 ± 0.1	5.5 ± 0.9	*No binding*
10	**A4**	6.6 ± 0.1	9.8 ± 2.8	*No binding*
11	**A5**	1.1 ± 0.2	2.2 ± 0.4	*No binding*
12	**A6**	4.3 ± 1.1	6.5 ± 1.2	*No binding*
13	**A7**	4.0 ± 0.9	4.3 ± 0.5	*No binding*
14	**I1**	6.5 ± 1.1	9.6 ± 1.5	*No binding*
15	**I2**	2.5 ± 0.6	5.5 ± 0.9	*No binding*
16	**I3**	4.0 ± 0.5	9.9 ± 4.5	*No binding*
17	**I4**	3.2 ± 0.7	>15	*No binding*
18	**BO1**	2.2 ± 0.3	2.7 ± 0.6	*No binding*
19	**BT1**	*No binding*	*No binding*	*No binding*
20	**TP1**	3.4 ± 0.8	8.3 ± 1.6	*No binding*

^1^ *K*_d_ values measured by tryptophan assay.

## Data Availability

The data presented in this study are available upon request to the corresponding author.

## Supplementary Materials

The following are available online at https://www.mdpi.com/article/10.3390/molecules27103097/s1, Figure S1: Superposition of X-ray vs. Hip312 best pose GDP, Figure S2: Superposition of X-ray vs. His312 best pose GDP, Figure S3: Best poses of the three singletons selected from the His312 grid, Figure S4: Tyr308-fragment centroids distances monitored during MD simulations. Figure S5: STD-NMR of **B1** with Rel*_Seq_* (1–385), Figure S6: STD-NMR of **A2** with Rel*_Seq_* (1–385), Table S1: Databases used for the virtual screening campaign, Table S2: 3D Datasets used in the VS workflow and enrichment factors calculated for each chemotype, Table S3: Fragment datasets obtained by using PubChem database, Table S4: Docking results for the fragment sets into HIP312 and HIS312 models.
