# Supplementary material for: New Chemotypes for the Inhibition of (p)ppGpp Synthesis in the Quest for New Antimicrobial Compounds
Source: Molecules. 2022 May 12;27(10):3097. doi: 10.3390/molecules27103097 (PMC9143738; doi:10.3390/molecules27103097)
Supplement: Supplementary file 1 [file molecules-27-03097-s001.zip › molecules-1713304-supplementary.pdf]

Supplementary Materials

New Chemotypes for the Inhibition of (p)ppGpp Synthesis in the Quest for New Antimicrobial Compounds

1. Supplementary Tables

Table S1. Databases used for the virtual screening campaign.

Fragment library	Number of 2D Molecules	Number of Generated 3D Molecules
Maybridge Rule of 3	2500	3036
Asinex Fragments	21,872	47,892
Life chemicals Fragment Libraries with Experimental Solubility Data I	11,667	20,204
Life chemicals Fragment Libraries with Experimental Solubility Data II	2921	6299
OTAVA Solubility fragment library	1021	1606
Chembridge Fragment library	13,808	28,037
SPECS Preplated fragment-based library	4532	7892
TOTAL	58,321	114,966

Table S2. 3D Datasets used in the VS workflow and enrichment factors calculated for each chemotype.

	His312 Grid		Hip312 Grid	
**Input structures**	114,966		114,966	
**Docking outputs after state penalty filter**	86,300		84,676	
**Duplicates removed**	74,226		72,617	
**Y308 aromatic interaction filter**	30,982		31,851	
		**Top 1% (EF)**		**Top 1% (EF)**
**CANVAS PAINS filters**	30,126	301	30,960	310
**Benzimidazoles**	1.1%	16.6% (**15.1**)	1,0%	6,1% (**6.1**)
**Amino benzoic acids**	0.2%	0.7% (**3.5**)	0,3%	6,5% (**21.7**)
**Indoles**	0.7%	2.0% (**2.9**)	0,8%	3,2% (**4.0**)

2. Supplementary Methods

Libraries expansion

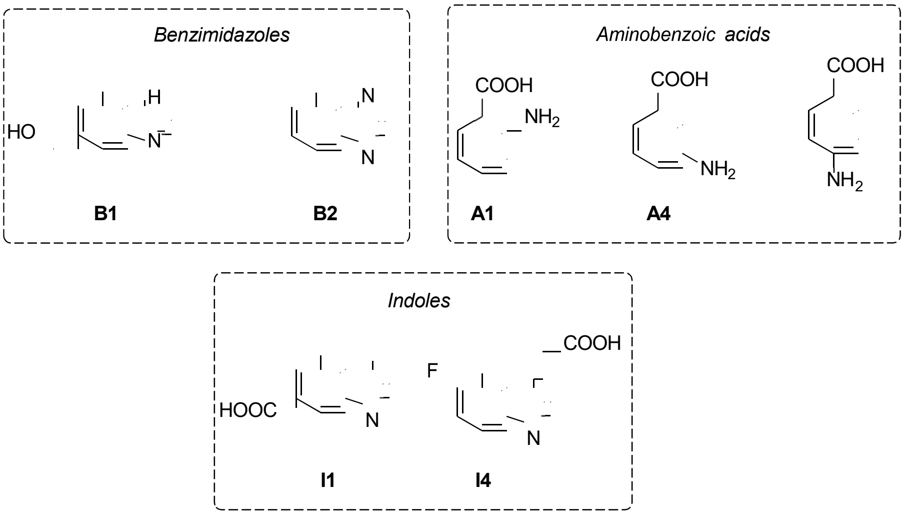

Starting from the fragments shown above, we performed a library expansion on the Pubchem database [1] (https://pubchem.ncbi.nlm.nih.gov) in order to maximize the exploration of the chemical space (2D Tanimoto index ≥ of 90%). The structures retrieved from Pubchem (Supplementary Table 3) were collected into 3 datasets, i.e. indoles, benzimidazoles, and aminobenzoic acids. After the removal of duplicates, the corresponding 3D structures were generated using Ligprep and filtered according to the Epik state penalty value (<0.6 kcal/mol). The fragments were then evaluated into the active site of the SYNTH domain of both HIS312 and HIP312 protein models using the VS docking protocol.

For each data set, docking results were analyzed and filtered saving the fragments able to form the aromatic interaction with Tyr308. A second step of duplicate removal was also performed saving the tautomer with the best docking score for each fragment. Three compounds **B4, B3** and **I3**, with improved or comparable ligand efficiency (Supplementary Table 4) to the parent VS fragments were identified and added to the selection. For the aminobenzoic acid set, fragment **A7** was available in the laboratory and was included in the study. For theses fragments, the stability of the interaction with Tyr308 was also confirmed over ten docking poses (i.e. formed by at least 5 poses out of 10).

Table S3. Fragment datasets obtained by using PubChem database. * 2D tanimoto similarity > 90%.

Query Fragment	n° of Downloaded Fragments *	n° of Fragments per Datasets	N° of Unique Fragments (Duplicate Filter)	N° of 3D Structures (Ligprep)	State Penalty Filter < 0.6 kcal/mol
**B1**	527				
**B2**	1593	2120	2049	7791	4023
**A1**	890				
**A4**	662				
**4-aminobenzoic acid**	667	2219	1612	2003	1658
**I1**	1426				
**I4**	2258	3684	3291	5920	5137

Table S4. Docking results for the fragment sets into HIP312 and HIS312 models. Docking score (Gscore) and ligand efficiency values (l.e.) are reported.

Fragment	Gscore Score (HIP312)	l.e.	Gscore (HIS312)	l.e.
**B1**	−6.496	−0.591	−7.499	−0.682
**B2**	−7.186	−0.599	−7.081	−0.590
**B3**	−7.319	−0.610	−7.413	−0.618

**B4**	−8.992	−0.599	−8.387	−0.495
**A1**	−6.429	−0.643	−5.753	−0.575
**A2**	−6.529	−0.544	−5.526	−0.460
**A3**	−7.010	−0.637	−5.917	−0.538
**A4**	−6.499	−0.65	−5.752	−0.575
**A5**	−7.101	−0.592	−6.125	−0.510
**A6**	−6.155	−0.560	−6.037	−0.549
**A7**	−6.621	−0.552	−6.21	−0.468
**I1**	−6.942	−0.579	−6.052	−0.504
**I2**	−6.536	−0.545	−6.41	−0.534
**I3**	−6.778	−0.565	−6.617	−0.551
**I4**	−6.634	−0.474	−6.691	−0.478
**BO1**	−7.251	−0.604	−6.317	−0.526
**BT1**	−7.083	−0.590	−6.808	−0.567
**TP1**	−8.424	−0.562	−7.317	−0.488

3. Supplementary Figures

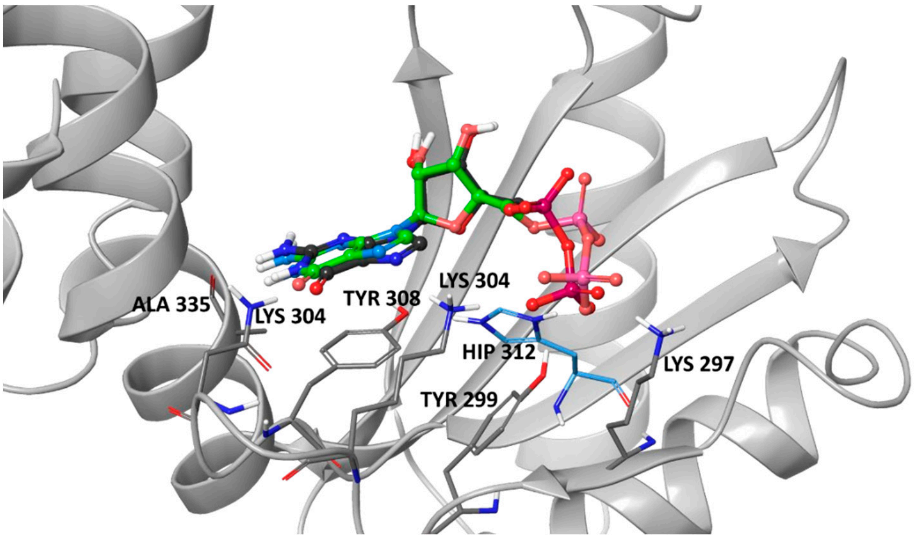

Figure S1. Superposition of X-Ray(grey) *vs* Hip312 best pose (green) GDP. The RMSD calculated on GDP atoms is 1.23Å.

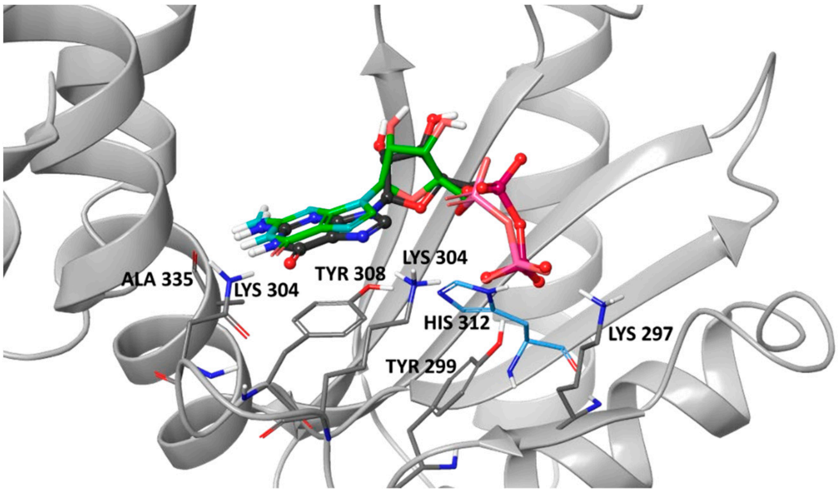

Figure S2. Superposition of X-Ray(grey) *vs* His312 best pose (green) GDP. The RMSD calculated on GDP atoms is 0.78Å.

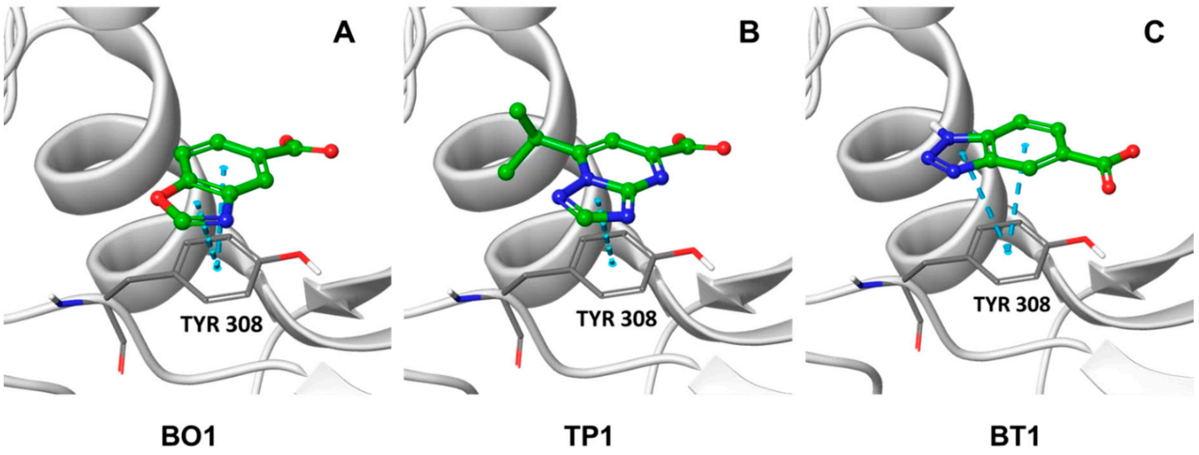

Figure S3. Best poses of the three singletons selected from the His312 grid. (**A**). boenzoxazole **BO1**; (**B**) triazolopyridine **TP1**; (**C**) benzotriazole **BT1.**

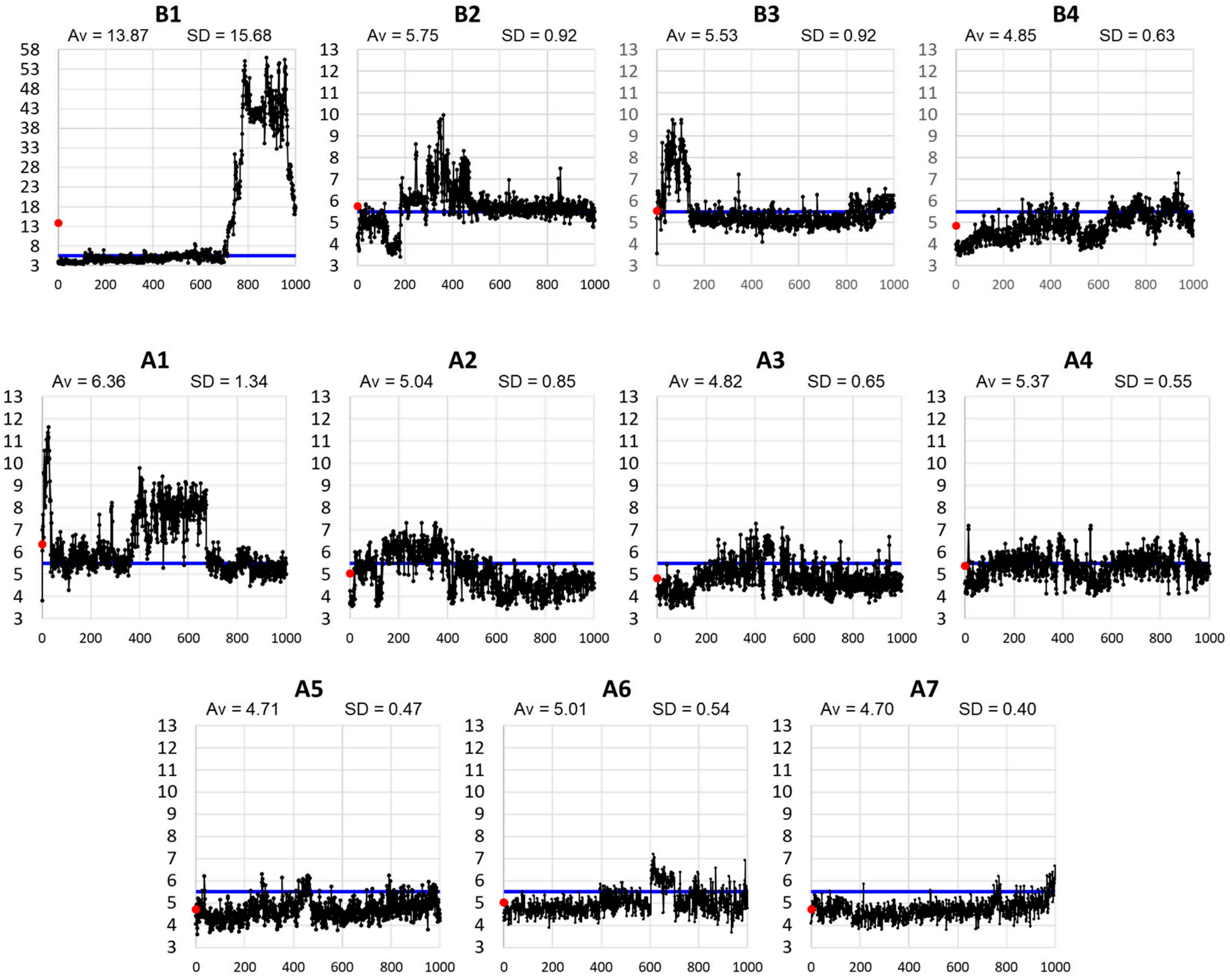

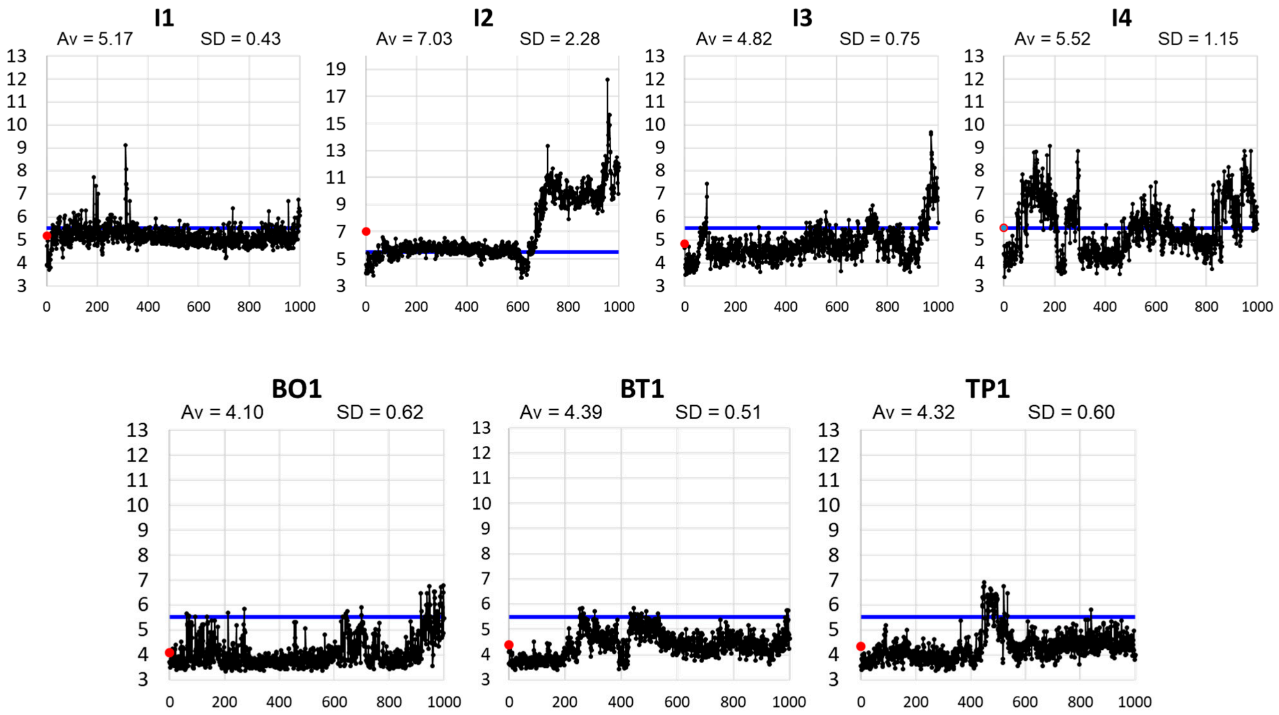

Figure S4. Distances between the centroids of Tyr308 side chain and of the fragment rings monitored during MD simulations (in black). The average (Av, red dots) and standard deviation (SD) of distances are also reported. The light-blue line defines the maximum distance at which a π-π interaction can be considered as formed.

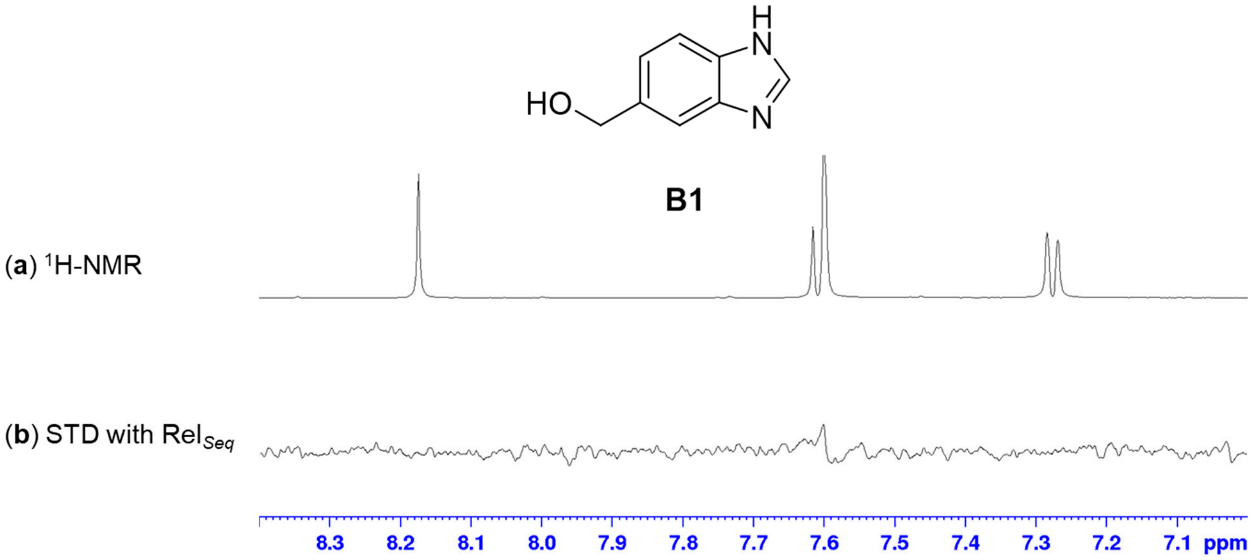

Figure S5. ^{1}H-NMR of fragment **B1** in phosphate buffer at 298K. (**b**) STD-NMR of **B1** with Rel*Seq* (1-385). Fragment : protein ratio 1000:1; fragment concentration 3mM.

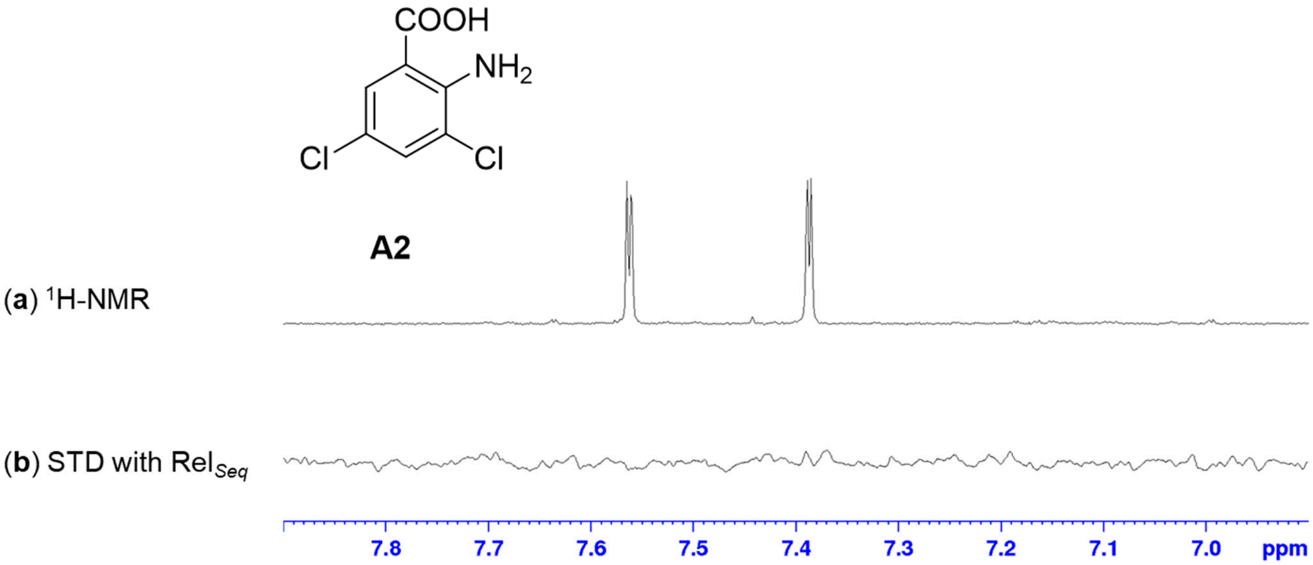

Figure S6. ^{1}H-NMR of fragment **A2** in phosphate buffer at 298K. (**b**) STD-NMR of **A2** with Rel*Seq* (1-385). Fragment: protein ratio 1000:1; fragment concentration 3mM.

References

1. Kim, S.; Chen, J.; Cheng, T.; Gindulyte, A.; He, J.; He, S.; Li, Q.; Shoemaker, B.A.; Thiessen, P.A.; Yu, B.; et al. PubChem in 2021: New data content and improved web interfaces. *Nucleic Acids Res.* **2020**, *49*, D1388–D1395.
